# Understanding the Use of Dietary Supplements among Athlete and Non-Athlete University Students: Development and Validation of a Questionnaire

**DOI:** 10.3390/sports7070166

**Published:** 2019-07-06

**Authors:** Dalia El Khoury, John J.M. Dwyer, Lindsay Fein, Paula Brauer, Sydney Brennan, Irene Alfaro

**Affiliations:** Department of Family Relations and Applied Nutrition, University of Guelph, Guelph, ON N1G 2W1, Canada

**Keywords:** dietary supplements, mixed-methods, reliability, theory of planned behaviour, validity, questionnaire

## Abstract

Background: The purpose of this study is to develop and test the validity and reliability of a questionnaire to evaluate dietary supplement use based on the Theory of Planned Behaviour (TPB). Methods: The questionnaire has sections on demographics, physical activity, dietary supplements, and cognitive constructs based on the TPB. Three stages are followed. In Stage 1, elicitation interviews are conducted on five varsity athletes, five physically active non-athletes, and five physically inactive University of Guelph (UofG) students. In Stage 2, comments and ratings of the TPB-based statements are gathered from 10 subject matter experts to check for content validity. In Stage 3, Cronbach’s α is calculated to determine the internal consistency of the cognitive constructs by a pilot test on 84 Applied Human Nutrition UofG students. Results: Interviews assisted in the formulation of the cognitive constructs’ statements, including intentions, attitudes, injunctive norms, descriptive norms, and perceived behavioural control. Content validity ensured that these constructs did not overlap. Few statements from the cognitive constructs were omitted based on findings from the reliability test, achieving acceptable Cronbach’s α values across all constructs (≥0.70). Conclusions: This supplement use questionnaire will be used in a future study to investigate the use and determinants of dietary supplements among Canadian athlete and non-athlete UofG students.

## 1. Introduction

The use of dietary supplements is increasingly reported worldwide, particularly among athletes, who use it to enhance athletic performance and improve exercise recovery [1,2,3]. In Canada, dietary supplements are categorized as Natural Health Products. According to Health Canada (2018), Natural Health Products are naturally occurring substances in a variety of forms that are used to restore or maintain good health [4]. In Canada, a high prevalence of supplement use was reported among athletes, including young athletes [1,3,5,6]; however, this was mainly explored in Alberta, with two studies from Quebec [7] and British Columbia [8]. Studies in other provinces, such as Ontario, are needed for a nationwide understanding of the status of dietary supplement use among athletes, particularly younger ones in university settings. 

Dietary supplementation is also becoming more prevalent in the general population. The use of dietary supplements has been commonly reported among physically active non-athletes in a number of studies [9,10,11,12,13,14,15]. This sub-population is attending gyms and exercising regularly in response to several types of marketing and other messages, including an increased optimal health focus and social pressure regarding physical appearance. 

This increased use of dietary supplements should not be ignored, considering its potential for toxicity, its potential for nutritional imbalances, and its association with the risk of doping. Cross-contamination with prohibited substances or undeclared ingredients on the label of dietary supplements has also been reported [16,17,18], reflecting an involuntary gateway to doping substances. In addition, the market for supplements has changed rapidly with the advent of internet marketing and selling, particularly among young adults.

A unique aspect of the present study is not only the investigation of dietary supplement use and the underlying reasons and sources of information among athletes and non-athletes in a university setting in Ontario, but also the incorporation of the Theory of Planned Behaviour (TPB) to explore the determinants of this use. The TPB was designed to predict health behaviour change under volitional control [19]. In this theory, the most proximal variable predicting behaviour is the individual’s intention to perform a given behaviour. Intentions are influenced by attitudes towards the behaviour, subjective norms, and perceived behavioural control [20]. Subjective norms assess the perceived social pressure to perform or not perform a particular behaviour. Two types of subjective norms are assessed in the TPB: descriptive norms and injunctive norms [21]. Injunctive norms are the perceived social pressures from significant others to perform the behaviour, whereas descriptive norms are the perception of whether other people perform the given behaviour. Perceived behavioural control refers to individuals’ evaluation of their own ability to perform this behaviour [20]. To our knowledge, research on the use of dietary supplements using the TPB is limited in Canada. A study in Quebec [7] examined the use of performance-enhancing substances in young athletes using this theory. 

The TPB appears to offer a useful framework for understanding or predicting behaviours based on psychological constructs generally theorized to influence individuals’ behaviour. Athletes’ psychosocial environments were found to have a significant impact on their decision to use performance-enhancing substances in a study by Goulet et al. (2010) [7]. Understanding the motivations, perceived beliefs, and contributing factors regarding the use of dietary supplements is essential, for the design of more effective messages regarding the pros and cons of using these supplements. It seems that communicating reliable information about health-conscious behaviour is not enough to induce significant behavioural changes in a population [7]. 

In the current article, we describe the development, validity, and reliability testing of a newly adapted comprehensive supplement use questionnaire that will be used in a larger scale study to assess the use of, beliefs about, and experiences with dietary supplements among athlete and non-athlete students in a university setting in Ontario.

## 2. Materials and Methods

### 2.1. Supplement Use Questionnaire

The questionnaire included four sections: physical activity patterns, dietary supplements, cognitive constructs based on the TPB, and demographics. The format of the questionnaire consisted of multiple-response questions, 1 to 5 Likert-type rating scales, and open-ended questions. 

In the first section, physical activity level was assessed using six questions from the short-form of the widely used international physical activity questionnaire (IPAQ, 2002; https://sites.google.com/site/theipaq/). The six questions assess the frequency (days per week) and duration (minutes per day) of three types of physical activity during the last seven days: vigorous physical activity, moderate physical activity, and walking. According to the IPAQ scoring protocol, metabolic equivalent (MET) values of 8.0, 4.0, and 3.3 were assigned to each activity, respectively. Total MET minutes per week were calculated. Based on the MET values, individuals were also classified as less physically active (<1000 MET minutes per week) or more physically active (≥1000 MET minutes per week). A cut-off value of 1000 MET minutes per week, equivalent to double the minimal physical activity recommendations for health, was reported to be associated with significant health benefits [22]. 

Questions in the second section were on the types and frequency of use of dietary supplements, as well as the reasons for use and sources of information, adapted from a previous study by El Khoury and Antoine-Jonville (2012) [10]. In the present study, dietary supplements listed in the questionnaire did not comprise prohibited substances by the world anti-doping agency (WADA) (2019) [23], such as anabolic steroids and growth factors. 

The third section was based on statements related to the different cognitive constructs of the TPB, including intentions, attitudes, subjective norms (injunctive and descriptive norms), and perceived behavioural control, with respect to the use of dietary supplements. The statements were based on a consensus process initiated by Fishbein and Ajzen (1975) [24] on the TPB. 

### 2.2. Protocol

A draft of the supplement use questionnaire was prepared, with the addition of questions addressing different TPB constructs. Three sequential steps were taken to further develop the questionnaire, particularly the five TPB sub-scales. In Stage 1, an elicitation study was conducted to elicit beliefs, attitudes, and norms regarding dietary supplement use. In Stage 2, ratings and comments were obtained from subject matter experts on the draft questionnaire’s content. In Stage 3, the revised questionnaire was administered to undergraduate and graduate Applied Human Nutrition students at the UofG to assess its reliability. For all three steps, the questionnaire and the study protocol were approved by the Research Ethics Committee of UofG. 

#### 2.2.1. Stage 1: Elicitation Study 

The first step, an elicitation study following a recommended protocol [25,26], aimed at the development of statements that accurately assess each of the cognitive constructs of the TPB. In total, 15 students were recruited, consisting of five participants from the three different categories of physical activity: athletes, physically active non-athletes, and physically inactive. This categorization reflected the nature of the planned sample in the larger study. With this sample of individuals representative of the target population, data saturation was achieved [26]. Individual, face-to-face interviews were conducted, each lasting approximately 30 minutes. During the interviews, questions were asked to elicit salient beliefs on supplementation practices. Some examples of the questions asked included: “What do you consider to be the advantages (and disadvantages) of you using dietary supplements?” (attitudes); “Which individuals or groups are most likely (and least likely) to use dietary supplements?” (descriptive norm); “Which individuals or groups would approve (and disapprove) of you using dietary supplements?” (injunctive norm); “Which factors or circumstances would make it easy or enable you (and difficult or prevent you) to use dietary supplements?” (perceived behavioural control). The interviews were audio-recorded and transcribed, and questions in the “cognitive construct” section of the questionnaire were developed based on themes identified in the scripts of the interviews. The draft questionnaire consisted of five statements for each of the attitude, subjective norm (i.e., descriptive norm and injunctive norm), and intention constructs and six statements for the perceived behavioural control construct. 

#### 2.2.2. Stage 2: Content Validation 

Content validity was examined by receiving feedback from a panel of subject matter experts, who are individuals with deep understanding, knowledge, and expertise in a particular area or topic. They evaluated the questionnaire for 12 factors, including brevity, length, number of items, clarity, repetition, order, precision of language, response options, scale, bias, double-barreled questions, and appropriateness of questions. These factors were suggested to be reasonable to assess when conducting content validation of a questionnaire [27]. In addition, participants rated the relevance of each statement in the “cognitive construct” section to the construct that the sub-scale intends to measure on a 1–3 Likert scale (i.e., 3 = very relevant; 2 = somewhat relevant; 1 = not relevant). These ratings and comments were incorporated into the questionnaire from Stage 1. 

#### 2.2.3. Stage 3: Reliability Test 

The reliability of the revised questionnaire was tested on 84 out of the 100 targeted undergraduate and graduate students enrolled in the Applied Human Nutrition program at UofG. This sample of 84 participants was considered adequate because of the strong Cronbach’s alphas of almost all cognitive constructs that were achieved, even before deletion of some of the cognitive constructs’ statements. Students completed an online version of the questionnaire through the Qualtrics software. Cronbach’s alpha was calculated for each set of statements within each of the five components of the “cognitive construct” section to examine internal consistency. George and Mallery (2003) provided the following rules of thumb for the classification of Cronbach’s alpha values: “>0.9 – excellent, >0.8 – good, >0.7 – acceptable, >0.6 – questionable, >0.5 – poor, and <0.5 – unacceptable” [28] (p. 231). Cronbach’s alpha values were used to revise the questionnaire from Stage 2. 

### 2.3. Data Analyses 

In Stage 3, statistical analyses were conducted using SPSS software (version 27.0, IBM Corp, Armonk, NY, USA). Descriptive statistics (mean and standard deviation) were used to describe continuous variables, and frequency statistics (number and percentage) were used to describe categorical variables. Self-reported body mass index (BMI) was calculated as weight (kg)/height (m)^2^. In addition, BMI was classified as underweight (<18.5 kg/m^2^), normal weight (18.5–24.9 kg/m^2^), and overweight/obese (≥25.0 kg/m^2^) [29]. In the regression analysis, we sought to identify predictors of supplement use. Since there was a small number of supplement non-users (n = 10) versus users (n = 74) in our sample, supplement use was computed as a continuous outcome variable reflecting the number of types of supplements consumed by participants. The unstandardized and standardized β values, p values, and 95% confidence intervals were generated for each of the different general characteristics by using univariate linear regression test to establish the degree of association between these potential predictors and the use of dietary supplements. All categorical variables were transformed to dichotomous variables, assigning a value of 1 to the most frequent sub-category and a value of 0 to all other sub-categories. Height, BMI, and BMI category variables were not analyzed as potential predictors of dietary supplement use due to the relatively small number of respondents who reported their height (n = 17). All variables that had p < 0.05 in the univariate analysis were fitted into a stepwise multivariate linear regression model. The R^2^ and the p value of the different models were computed, reflecting how close the data were to the fitted regression model. A p value < 0.05 was considered statistically significant.

## 3. Results 

### 3.1. Participant Characteristics 

#### 3.1.1. Stage 1: Elicitation Study 

A total of 15 undergraduate students participated in Stage 1 of the study. All participants were female. The average ages of the participants were 21 for physically active athletes, 21 for physically active non-athletes, and 20 for physically inactive individuals.

#### 3.1.2. Stage 2: Content Validation

Eleven subject matter experts were contacted. Ten of them, including M.Sc. student/student investigators (n = 2), applied social psychology researcher (n = 1), applied human nutrition researchers (n = 3), human health and nutritional sciences researchers (n = 2), exercise physiologist (n = 1), and registered dietitian (n = 1), agreed to participate in Stage 2 of the study. 

#### 3.1.3. Stage 3: Reliability Test

Of the 84 students, most participants were female (96.4%) and in the 21–23-years category (59.8%), with an average age of 22 years old. Most of the participants were physically active non-athletes (80.2%) versus physically active athletes (6.2%) and physically inactive individuals (13.6%). 

### 3.2. Questionnaire Development

#### 3.2.1. Stage 1: Draft Questionnaire 

In this stage, statements within each of the TPB cognitive constructs were formulated based on the scripts of the interviews. Examples of these statements are “In the next 6 months, I intend to take or keep taking a dietary supplement to improve my performance or general health.” (intention); “I believe that using dietary supplements may improve my physical appearance.” (attitude); “Among your friends, how often have you heard about someone engaging in supplement use?” (descriptive norm); “My teammates or training partners would support my use of dietary supplements to improve my performance or general health.” (injunctive norm); and “The price of dietary supplements would influence my use.” (perceived behavioural control). 

#### 3.2.2. Stage 2: Content Validity 

In this stage, comments on the questionnaire developed in Stage 1 and ratings for the cognitive construct statements were gathered from subject matter experts and then used to further revise the questionnaire. 

Ratings were gathered on whether each statement in the cognitive construct section of the questionnaire was relevant to the construct being tested (e.g., 3 = very relevant; 2 = somewhat relevant; and 1 = not relevant). Overall, median of the ratings of the different constructs were 3 (i.e., very relevant) (Table 1). 

Significant changes to the questionnaire were made. Some of the revisions included increasing the clarity of questions, revising the order of questions; requesting training hours per week from the participating varsity athletes, placing the demographics section at the end of the questionnaire, and including “prefer not to disclose” for sensitive questions. In addition, the supplement use section was revised by asking participants to report how long, how often, and the reason(s) for taking each of the selected supplements rather than supplements overall. The wording “prohibited supplements” was replaced with “non-dietary supplements” because it did not emphasize by whom it was prohibited. The corresponding question was placed at the end of the supplement use section, since the scope of the questionnaire is not the exploration of WADA-prohibited substances.

A number of comments were, in particular, made on the intention and perceived behavioural control constructs. Changes were applied accordingly to these constructs. The intention construct had comments on the wording used and the overlapping among statements. For example, in the statement “In the next 6 months, I want to take or keep taking a dietary supplement to improve my performance or general health.” one may think you want to perform a behaviour (i.e., exercise) but still not intend to do it. This statement was evaluated to be unnecessary and was removed. The perceived behavioural control construct had comments on the wording used and the double-barreled statements. For example, the wording “would” was replaced by “is” to reflect a present term. An example of a double-barreled statement is “I believe that using dietary supplements may be a safe, evidence-based way to improve my performance or energy.”. Beliefs that the use of dietary supplements will improve performance (i.e., the efforts made to achieve specific objectives) or “energy” (i.e., the strength and vitality required for sustained physical or mental activity) are two different concepts. This issue was addressed by removing energy so that the statement reads, “I believe that using dietary supplements will improve my performance.”.

#### 3.2.3. Stage 3: Reliability of the Questionnaire 

The revised draft from Stage 2 was additionally revised based on the reliability test completed in a pilot test on 84 Applied Human Nutrition UofG students. In order to achieve the most acceptable Cronbach’s α values across all constructs, a total of four statements were removed from the cognitive construct section, including 1 intention statement, 2 attitude statements, and 1 perceived behavioural control statement (Table 2). After the deletion of the four statements, all of the cognitive constructs had acceptable, good, or excellent Cronbach’s α values except for attitude in which the highest possible Cronbach’s α value was achieved. 

### 3.3. Supplement Use Patterns 

#### 3.3.1. Rate of Dietary Supplement Use

Of the 84 participants, 88.1% (n = 74) reported the use of one or more dietary supplements in the past 6 months. All males (100%, n = 3) and most females (87.7%, n = 81) consumed one or more dietary supplements. Most 18–20-year-olds (57.1%, n = 14) and 21–23-year-olds (93.9%, n = 49) and all of 24–26-year-olds (100%, n = 9) and > 27-year-olds (100%, n = 10) consumed one or more dietary supplements. Based on physical activity categories, all physically active athletes (100%, n = 65), most physically active non-athletes (87.7%, n = 11), and most physically inactive individuals (81.8%, n = 5) consumed one or more dietary supplements. Finally, based on sport type (if a varsity athlete), all endurance athletes (100%, n = 1) and power/strength athletes (100%, n = 2) and no intermittent athletes (0%, n = 1) consumed one or more dietary supplements. 

#### 3.3.2. Types of Dietary Supplements 

The most commonly used dietary supplements were vitamins/minerals, protein, fatty acids, prebiotics/probiotics, and carbohydrates. One hundred percent of participants who used dietary supplements in the past 6 months had used vitamins/minerals (Table 3).

#### 3.3.3. Sources of Information

The most commonly used sources of information on dietary supplements were health care professionals (e.g., physicians, team physicians, specialists, dietitians, and sports nutritionists) (81.1%), my own judgment (73.0%), the internet (67.6%), friends/family (48.6%), and other(s) (28.4%). 

#### 3.3.4. Predictors of Dietary Supplement Use

In univariate linear regression, five predictors were significant. Specifically, age (standardized β = 0.26; p = 0.017), body weight (standardized β = 0.29; p = 0.009), gender (standardized β = −0.22; p = 0.045), university major (standardized β = −0.24; p = 0.030), and medical condition (standardized β = −0.28; p = 0.010) were significant predictors (Table 4). Regarding positive predictors, being older and having a larger body weight were associated with higher dietary supplement use. Regarding negative predictors, being a female, enrolled in the Bachelor of Applied Science program (compared to other program majors), and not having a physician-diagnosed medical condition were associated with lower dietary supplement use. 

## 4. Discussion

To our knowledge, our focus on the TPB as a model to explore the use of dietary supplements among both athlete and non-athlete university students is relatively unique in Canada. In this study, a supplement use questionnaire was developed, assessed for face validity, and tested for inter-item reliability in the target population of university athlete and non-athlete students. 

Studies on the use of dietary supplements have generally implemented previously designed questionnaires without retesting their validity and reliability. Erdman et al. [1,5], in their studies on dietary supplementation in high-performance Canadian athletes, used a standardized questionnaire developed, validated, and tested for reliability by various health professionals, a sport administrator and researchers associated with the University of Calgary and the Canadian Sport Centre-Calgary. On the other hand, Wiens et al. (2014) [3] modified a questionnaire used by Erdman et al. (2006) [1] and checked its validity and reliability in their target population of high-performance Canadian athletes. As the questionnaire was directed towards a younger population, the language and format were adjusted. As well, since Wiens et al. (2014)’s study was a more recent study, additions were made to the list of dietary supplements, and electronic means of education were added [3]. 

Based on findings related to the elicitation study, content validity, and Cronbach’s alpha values, our supplement use questionnaire appeared to be a valid and reliable tool to implement on university students irrespective of their physical activity habits. 

Reliability was evaluated by assessing the internal consistency of the questionnaire, particularly the cognitive construct section, reflected by the Cronbach’s alpha values. This reliability testing method was different from ones used in other studies in Canada exploring dietary supplement use; test and retest as well as Kappa coefficient tests were used to assess the reliability of their questionnaires [1,3]. However, these studies did not explore the cognitive determinants of supplement use based on TPB constructs. Studies that used the TPB to assess different health-related behaviours used the Cronbach’s alpha test to measure reliability [7,30,31].

With respect to feasibility and respondent burden, the online format allows respondents to skip some sections, while prompting them to consider all types of supplements compared to open-ended questions. With 121 individual items and four distinct subsections, it took participants approximately 25 minutes to complete the questionnaire. Further work is needed to see if the length of the questionnaire can be reduced.

In the present study, 100% of athletes reported the use of one or more dietary supplements in the past 6 months. Therefore, our findings confirm that young athletes, in Ontario as in other provinces, rely on dietary supplements to support different sports-related performances. An earlier study that examined dietary supplementation of elite level athletes in Canada found that most (88.4%) had used one of more dietary supplements in the last 6 months [1]. A recent study conducted by Parnell et al. (2016), on young Canadian athletes, found that 100% of athletes used some type of dietary supplement at least once in the past 3 months [6]. The use of dietary supplements was also shown to be increasingly prevalent in the general population. In the pilot study by Parnell et al. (2016), the majority of non-athlete university students (87%) were also found to use dietary supplements [6]. According to the British Columbia Ministry of Health Planning (2004), 52% of men and 62% of women aged 19 to 30 reported using at least one supplement during the last month [32]. In addition, El Khoury and Antoine-Jonville (2012) found that the prevalence of supplement use among gym exercisers in Beirut city, Lebanon was 36.3% [10]. 

Vitamins/minerals, protein, fatty acids, prebiotics/probiotics, and carbohydrates by both athletes and non-athletes were the most commonly used dietary supplements. One hundred percent of participants who used dietary supplements have used vitamins/minerals. Similarly, other studies have shown that young Canadian athletes most frequently consume multivitamins, multi-minerals, vitamin C, vitamin D, sports bars, and protein powders [6]. Furthermore, young gym exercisers in Beirut city most commonly consumed protein power, amino acid pills, whey protein, creatine, and multivitamins [10]. 

In the current study, health care professionals were the most commonly used sources of information on dietary supplements, followed by my own judgment, the internet, friends/family, and other(s). Therefore, there is still some dependence on unreliable sources but lower than reported in other studies [2,3,5]. However, it is important to note that participants in the reliability test were students completing a degree in applied human nutrition and because of this these students may have had some prior exposure to knowledge on dietary supplements and their impact on health through their university program. The increased dependence on unreliable sources of information about dietary supplements may lead to decreased awareness about the health risks and contamination concerns [3]; this needs to be thoroughly addressed through the design and dissemination of appropriate and individualized nutrition education materials. 

The present study has several strengths. A key advantage is that, to our knowledge, this is the first questionnaire developed, validated, and tested for reliability to assess dietary supplement use among Ontario university students of different physical activity patterns, including athletes, physically active non-athletes, and physically inactive individuals. As well, to our knowledge, this is the first questionnaire developed to explore the psychosocial determinants of supplement use in addition to use, reasons for use, and sources of information. 

The main limitation of this study is the potential for response bias when receiving self-reported data via personal recall in Stage 3. Participants, especially athletes, might decide not to report on their dietary supplement use for a number of reasons. Athletes might not report the use of prohibited or non-prohibited supplements if they perceive the consequences of a positive doping test as unacceptably high. In addition, there are cultural and religious norms, moral values, and social pressure from close relatives and friends against the use of supplements [33]. Possibly, participants are consuming dietary supplements without the consent of their parents, guardians, or coaches, and they do not wish to reveal that information with the risk that this information may become released. Another limitation is the relatively poor Cronbach’s α value of 0.54 for the attitude’s construct. However, this value was considered acceptable as we did not want to remove more statements from this construct to ensure cohesiveness among the constructs with respect to the number of statements. Furthermore, the fact that 100% of participants in stage 1 and 96% of participants in stage 3 were females is a limitation worth mentioning. However, gender is not expected to have a major impact on information gathered during the elicitation interviews, and on data collected with respect to the internal consistency of cognitive constructs. In addition, considering the nature of the questions, gender is not expected to impact the generalizability of the questionnaire to all genders.

This study encompassed the development of a questionnaire tailored to determine dietary supplement use based on the TPB among young university athletes and non-athletes in Ontario. Findings from this study as well as other future studies using this questionnaire may contribute to a better understanding of the status and determinants of supplement use in the younger population of Ontario, irrespective of their physical activity patterns, and may inform health care professionals to design effective educational materials on dietary supplements, their benefits, and their risks. Further research that examines the additional validity of the questionnaire and test-retest reliability is warranted.

## Figures and Tables

**Table 1:** Ratings—Stage 2—of the cognitive construct section of the questionnaire.

Cognitive Construct	Median of Ratings
Intention	3
Attitude	3
Injunctive norm	3
Perceived behavioural control	3
Descriptive norm	3

**Table 2:** Internal consistency—Stage 3—of each set of the cognitive constructs of the questionnaire.

Cognitive Construct	Cronbach’s α Before Deletion of Statements (21 statements)	Cronbach’s α After Deletion of Four Unsuitable Statements (17 statements)
Intention	0.965	0.957
Attitude	0.540	0.542
Injunctive norm	0.831	0.831
Perceived behavioural control	0.788	0.743
Descriptive norm	0.822	0.822

**Table 3:** Types—Stage 3—of supplements used by dietary supplement users (n = 74).

Types of Supplements	n (%) of Dietary Supplement Users
Vitamins/minerals	74 (100%)
Protein	47 (63.5%)
Fatty acids	29 (39.2%)
Prebiotics and probiotics	27 (36.5%)
Carbohydrate	19 (25.7%)
Herbs and botanicals	14 (18.9%)
Other unlisted supplement(s)	11 (15.3%)
Stimulants/energy boosters	10 (13.5%)
Amino acids	8 (10.8%)
Digestive enzymes	8 (10.8%)
Non-vitamin/mineral antioxidants	6 (8.2%)
Fat burners/weight loss	5 (6.8%)
Meal replacements/weight gainers	3 (4.1%)
Nitrates, nitric oxide, ‘pump’, and vasodilators (e.g., beetroot juice or powder, l-arginine, and citrulline malate)	3 (4.1%)

n = sample size.

**Table 4:** Association—Stage 3—between continuous and categorical variables and dietary supplement use in univariate linear regression model (n = 84).

Variable	Unstandardized β	Standardized β	P Value	95% CI
Continuous variables				
Age (years)	0.18	0.26	0.017*	0.03–0.32
Weight (kg)	0.06	0.29	0.009*	0.02–0.11
MET (minutes/week)	0.00000577	0.02	0.887	0.00–0.00
Categorical variables				
Gender	−2.40	−0.22	0.045*	−4.73–0.06
Ethnicity	0.98	0.20	0.064	−0.06–2.02
University major	−1.17	−0.24	0.030*	−2.22–0.12
Parent’s education	−0.14	−0.03	0.764	−1.08–0.79
Smoking status	−0.87	−0.11	0.315	−2.59–0.84
Alcohol intake	−0.65	−0.14	0.203	−1.65–0.36
Medical condition	−1.25	−0.28	0.010*	−2.20–0.31
Physical activity category	0.71	0.15	0.161	−0.29–1.71

*p < 0.05.; n = sample size; CI = confidence interval.
